# Food Security in the Rural Mapuche Elderly: Analysis and Proposals

**DOI:** 10.3390/nu16234042

**Published:** 2024-11-26

**Authors:** Angélica Hernández-Moreno, Olga Vásquez-Palma, Leonardo Castillo-Cárdenas, Juan Erices-Reyes, Alexsa Guzmán-Jiménez, Carlos Domínguez-Scheid, María Girona-Gamarra, Marco Cáceres-Senn, Jorge Hochstetter-Diez

**Affiliations:** 1 Departamento de Salud Pública, Facultad de Medicina, y Centro de Estudios y Promoción de los Derechos Humanos, Universidad de La Frontera, Temuco 4811240, Chile; angelica.hernandez@ufrontera.cl; 2 Departamento de Procesos Terapéuticos, Universidad Católica de Temuco, Temuco 4813302, Chile; 3 Departamento de Ciencias Jurídicas, Facultad de Ciencias Jurídicas y Empresariales, Temuco 4811240, Chile; leonardo.castillo@ufrontera.cl (L.C.-C.);; 4 Centro de Estudios del Desarrollo, Providencia 7500026, Chile; alexsa.guzman@gmail.com; 5 Doctorado en Derecho, Ciencia Política y Criminología, Universidad de Valencia, 46010 Valencia, Spain; dominguezscheid@gmail.com; 6 Escuela de Nutrición, Departamento de Nutrición Básica, Universidad de la República, Montevideo 11600, Uruguay; mgirona@nutricion.edu.uy; 7 Carrera de Ingeniería Informática, Facultad de Ingeniería y Ciencias, Universidad de La Frontera, Temuco 4811240, Chile; m.caceres04@ufromail.cl; 8 Departamento de Ciencias de La Computación e Informática, Facultad de Ingeniera y Ciencias, Universidad de la Frontera, Temuco 4811240, Chile; jorge.hochstetter@ufrontera.cl

**Keywords:** right to food, Mapuche elders, food security, rurality, public policies

## Abstract

Background: The increase in population longevity often occurs in contexts of inequity and relative poverty, accompanied by economic deterioration. This becomes a social determinant that has a direct impact on food security. This phenomenon particularly affects certain groups and territories, although there is still a lack of disaggregated references. Intersections between factors such as being a rural inhabitant, Indigenous, woman, or elderly person are observed in relation to food security, which forces us to pay greater attention to gaps that have remained invisible for years. Objective: The objective of this study is to analyze the main factors that affect the food security of Mapuche men and women over 60 years of age living in the rural area of Temuco, Chile. Method: Qualitative, descriptive, and interpretative research was carried out, observing the process from the interpretative symbolic paradigm and the complexity approach. Results: The data are made up of the discourses of these subjects, whose analysis allowed for the identification of results. These results show that producing their own food enables rural Mapuche elders to achieve food security. The cultural food heritage preserved by Mapuche elders, especially women, acts as a facilitating factor, as do community spaces that reinforce their culture. Among the obstacles to food security are migration to the city for work, pathological aging, and the limited production of culturally healthy foods (affected by environmental problems, cultural changes, the destabilization of group identity, and public policies that are incongruent with the territorial worldview). Conclusions: While rural Mapuche elders retain valuable practices for their food security, inadequate policies, migration and environmental degradation present significant challenges.

## 1. Introduction

One of the United Nations (UN) Sustainable Development Goals (SDGs), which seems far from being achieved, is to end hunger by 2023. Despite the fact that, as the Food and Agriculture Organization of the United Nations (FAO) points out, there is an excess of food produced, one in seven people in the world still go hungry [1]. This is primarily due to famine on the African continent, which is largely caused by frequent and worsening droughts that are becoming more common across all continents [2,3]. This is leading to greater inequality in access to healthy food. Another significant factor lies in the market, such as the sharp rise in agrifood prices, which mainly affects food-importing countries. This situation is exacerbated by the high concentration of natural resources under corporate control and the severe precarization of the peasantry [1].

The global disparities in access to healthy food represent a dehumanizing reality [4]. In other words, those responsible for these disparities deny the humanity of those affected. Therefore, it is essential to exert ethical pressure on governments and international organizations to eradicate these inequalities.

A total of 29.6% of the world’s population experiences moderate or severe levels of food insecurity, meaning they do not have access to adequate food [5]. This issue particularly affects vulnerable groups such as women, children, rural communities, Indigenous peoples, the elderly, and migrants [6].

The new United Nations report, Panorama of Food and Nutritional Security 2022, states that 22.57% of people in Latin America and the Caribbean do not have sufficient means to access a healthy diet. In the Caribbean, 52% of the population has been affected by this situation; in Mesoamerica, this number reaches 27.8%, and in South America, 18.4%. It was reported that 131.3 million people in the region could not afford a healthy diet in 2020. This represents an increase of 8 million with respect to 2019 and is due to the higher average daily cost of this type of diet in Latin America and the Caribbean compared to the rest of the world’s regions, reaching the Caribbean value of USD 4.23, followed by South America and Mesoamerica with USD 3.61 and USD 3.47, respectively [5].

In this paper, we will refer to a specific segment of the vulnerable groups, which are the rural Indigenous elderly. In this context, Chile presents an accelerated population aging process; 19.7% of its population was over 60 years old in 2020, being one of the four Latin American countries in an advanced stage of aging, and in 2050, it will be one of the most aged countries in the region, with 30.6%. In La Araucanía, this corresponds to 21.6% of the population [7]. Although this increased longevity is associated with improved living conditions, the extension of this stage is also accompanied by a rapid decline and deficits in access to basic social benefits, considering the lack of equity and economic precariousness of the aging population, which is an important social determinant that influences food security.

Addressing the aging of marginalized groups requires understanding its magnitude and implications, which is not always feasible due to the information gap regarding specific groups, such as the rural population, as highlighted in the literature review [8]. This study seeks to address this gap by investigating the perspectives of rural Mapuche elders on their food security in relation to culture, public policies, and implemented systems, and their role as either facilitating or limiting factors in the development of food security.

Aging and the inequalities that have become a pandemic pose enormous challenges for laws and public policies aimed at strengthening rights related to human welfare, especially within the framework of the Inter-American Convention on the Protection of the Human Rights of Older Persons, ratified by Chile [9]. In order to evaluate the effectiveness of these initiatives, it is necessary to have references that facilitate the planning of legislation and policies that account for the diverse realities of this population group. However, as previously mentioned, there is a lack of data on sensitive geographic areas, showing intersections between being a rural inhabitant, an Indigenous person, a woman, and an elderly person with respect to food insecurity. This highlights the need to focus more on the gaps that have been overlooked for years.

In this context, the increase in the age of inhabitants and the search for opportunities outside the territory have contributed to the problems faced by peasant and Indigenous family farming, making it difficult for them to continue to exist. Even though this sector has received very little support from public policies, it has contributed to a greater diversity of food for humans [10]. Today, young people migrate to big cities, and the elderly do their best to continue producing their own food, as they are practically discarded by the system. This has a negative impact on the life role of adults, the elderly, and rural woman, which is closely related to traditional agriculture, which provides sustenance to the family [11]. In addition, this undermines the right of the elderly to an adequate and varied diet. This is a matter of concern due to the importance of nutrition in this stage of life, which promotes better health, mental health, integration, and autonomy [12]. Studies in Latin America indicate that Indigenous food systems are based on the broad biodiversity of their ecosystems for food production and harvesting, which is related to cultural heritage and the reaffirmation of identities [13] and, on the other hand, that human groups with the lowest welfare conditions, Indigenous and rural populations, are more food insecure [14].

The objective of this study is to analyze the main factors affecting the food and nutritional security of Mapuche men and women over 60 years of age living in the rural area of Temuco in the years 2022 and 2023. Our contribution consists of gathering sensitive information regarding the perception of this age and ethnic group of rural inhabitants on the state of their food security and the factors that influence it.

## 2. Background

Food security is a multidisciplinary problem at the national and international levels, and in many countries it is a priority line of action, being an elementary condition for the welfare of the population, a point of support for strategic processes of participation, personal growth, education, determinant of nutritional status, and general health of a person, which in turn influences the physiological processes in general or the competence of people as workers, producers, and consumers; not to mention its influence on the economic, political, sociological, and environmental factors [15].

Food security was initially defined at the World Food Conference in 1974, relating it to the supply of basic foods at all times for the growing population, and this concept was later defined as food availability. Over time, it has become more complex. For example, in the Rome Declaration of 1996, the dimensions of people’s access to food and its biological utilization in the human body were incorporated. Later, in 2009, the dimension of stability of the previously mentioned factors was added, followed by cultural adequacy. In recent decades, the condition of sustainability has been included, referring to the ecosystem impacts of agriculture [16].

Likewise, its presence is measured by the distribution and income of sufficient, safe, varied, equitable, and apt food for consumption, immersed within an environment that guarantees the tranquility of the population to have access to it. But, from the point of view of epidemiology and public health, its definition is associated with the absence of disease or contamination with pathogenic organisms that cause structural or physiological complications at the systemic level [16]. Food security is understood to be achieved when people’s need for sufficient and safe food is satisfied. Initially, food security did not address the means or methods to achieve it, but concern has been growing about the environmental problems caused by food production within the framework of global challenges [16].

According to the World Health Organization (WHO), older adults are those who are 60 years of age or older. Globally, it is projected that this population sector will continue to grow and will exceed two billion by the year 2050. According to recent research on aging in Latin America, the average growth rate of older adults living in the countries of this region is 3.4 percent per year. This figure is growing faster than the number of children, underscoring the importance of aging as a social phenomenon. Older age by itself need not be synonymous with declining health [17]. Global aging has implied new, more integrative and multidimensional approaches, highlighting the context in which people age and focusing efforts on the basis of positive aging, on the maintenance of the physical, cognitive, social, and productive functions of the elderly [18].

On the other hand, 38.5% of Chile’s senior citizens (MS) state that their monthly income is less than CLP 200,000 [19], with 66% of this value being women; they suffer lower incomes due to longer life expectancy [20], a situation aggravated by the conditions imposed by the Chilean pension system [21]. One in three elderly people state that their income does not allow them to meet their basic needs, and 19% of elderly-only households are permanently worried about not having enough food [19]. The precariousness triggered by the COVID-19 emergency we are experiencing has focused on specific sectors and human groups. In the country, 10.8% of the population suffers from poverty, which increases to 13.8% in the rural sector, 13.2% in the Indigenous population, and 5.6% in the elderly, increasing multidimensional poverty to 22.1% [22].

In La Araucanía, one of Chile’s poorest regions, 17.4% of elderly people are in this condition, as are 37.3% of households with an elderly head of household [23]. These averages reflect realities in a country whose macroeconomic indicators appear to be one of the best in the region. In addition to the above, there is a strong and misguided tendency to approach this age group as if it corresponded to a homogeneous category that shares the same problems, attributes, and needs [24]. This greatly limits the possibilities to diversify and profile interventions according to personal resources, culture, territory, and specific needs.

## 3. Materials and Methods

Qualitative, descriptive, and interpretative research was carried out, observing the process from the interpretative symbolic paradigm [25] and the complexity approach [26]. Both theoretical places allow the observation and analysis of the subjectivity expressed by people to answer the research question: what are the factors that affect the food security of Mapuche people over 60 years old, particularly inhabitants of the rural area of Temuco?

**Figure 1:**
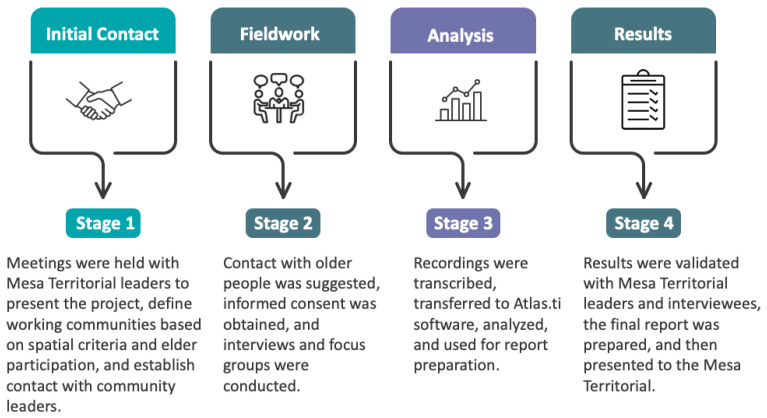
Work methodology.

This research was approved by the Scientific Ethics Committee of the Universidad de La Frontera in Evaluation Act N°075/22, dated 3 August 2022. Subsequently, fieldwork was carried out with a qualitative methodology with ethnographic support, with convenience sampling according to the objectives, which allowed the selection and participation of members of the territory. The methodology is divided into four stages (see Figure 1).

**Figure 2:**
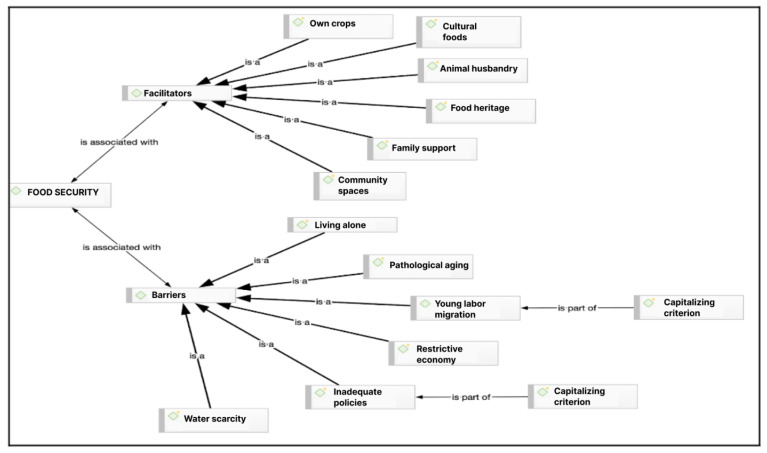
Barriers and facilitators of food security for Mapuche elderly in rural Temuco.

**Figure 3:**
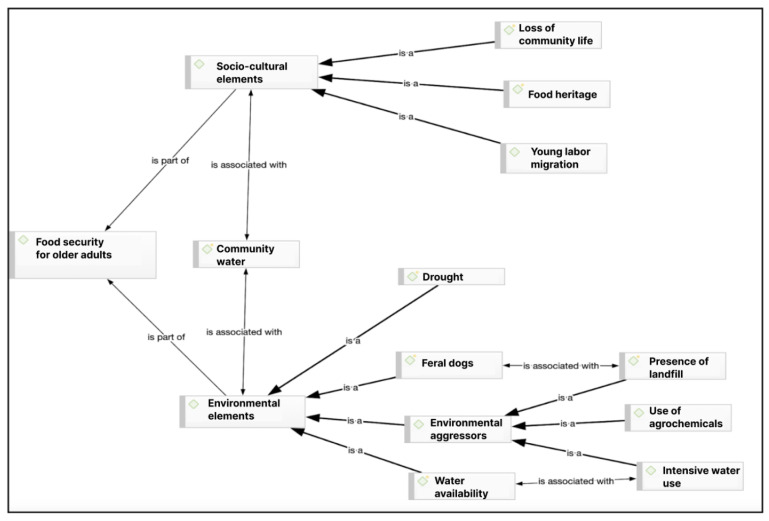
Sociocultural and environmental elements that affect food security among rural Mapuche older people in Temuco.

**Figure 4:**
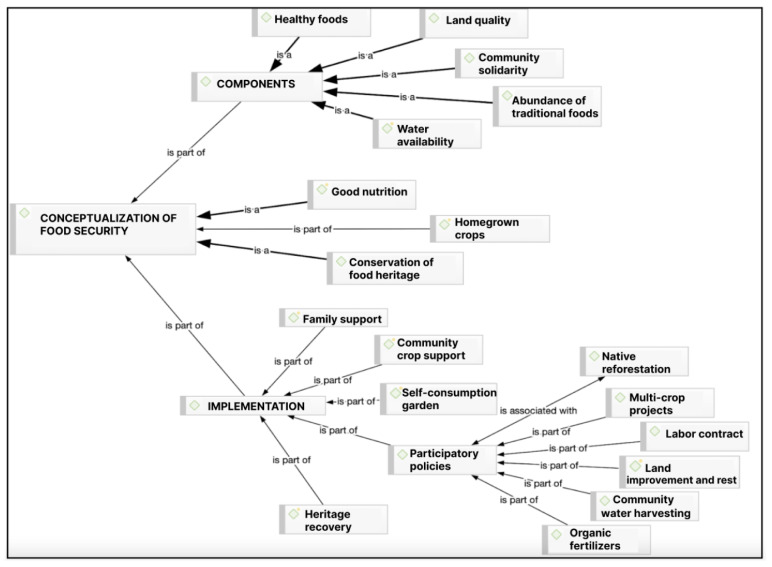
Conceptualization and components of food security and its implementation in rural areas.

(i) Stage 1: Contact with the territory. This was carried out through the Boyeco Territorial Roundtable. A meeting was held with leaders, after which the communities were informed of the project in an extended territorial meeting. To define the study participants, the territory was spatially divided into four segments (mapping), in which the communities to be intervened and the potential participants were identified.

(ii) Stage 2: Fieldwork and study participants. Fieldwork was conducted in nine Mapuche communities in Boyeco, Temuco, in the Araucanía Region of Chile. The people interviewed were men and women over 60 years of age, as well as leaders from the mentioned territory. The type of instrument applied was an ethnographic interview (Geertz, 2012), adapted to the subject and their contextual conditions, guided by the research objective. The richness of this type of interview is associated with its flexibility and the opportunity it provides participants to clearly and deeply express their ideas. For this reason, questions are approached based on the conceptual themes suggested by the objectives, and no closed or repetitive questions are pre-formulated for each interviewee. Eleven open interviews were conducted, each lasting an average of an hour and a half. As a complementary technique, two focus groups were conducted, each consisting of at least ten people, including both men and women who were members of Indigenous community organizations from the same territory. The duration of these sessions was approximately two hours. Both instruments were applied in different communities within the same territory.

(iii) Stage 3: Analysis of results. To obtain the results, an intersectional analysis of the data was carried out based on the grounded theory [27]. The process begins by linking the research objectives with the discourse obtained through interviews and focus groups and selecting relevant quotes. Emerging conceptual categories were constructed based on the discourse of the interviewees, considering their meanings and their particular worldviews in response to the objectives explored. Subsequently, more abstract ideas were developed and associated with existing theory, leading to the construction of conceptual networks (Figure 2, Figure 3 and Figure 4). This was supported by the Atlas/ti 22 software.

(iv) Stage 4: Communication of results. The results were presented to the participating communities and validated by them.

For the validity and limit of the application of the data construction techniques, we resorted to the application of the saturation phenomenon [28,29] and triangulation by the author and technique [30]. The research was carried out within a framework of ethical protection in accordance with bioethical deontological principles and current legislation in Chile.

## 4. Results

The results obtained in relation to each of the specific objectives proposed are presented below.

### 4.1. Food Safety Enablers and Barriers

Figure 2 presents the results regarding the facilitators and barriers to food security from the perspective of rural Mapuche elders interviewed. In the own crops code, they state that having the possibility of cultivating their fields and producing their own food allows them to have food security (quote interview 4, in Table 1).

Regarding so-called “cultural foods”, they state that growing foods with which they grew up and which were validated by their culture of origin are recognized as nutritious. This makes them feel that they have food security. Therefore, the lack of them generates a feeling of scarcity and food insecurity (quote from community leader 1, in Table 1).

Poultry and animal husbandry are equally important for the elderly. The raising of chickens of various breeds has been largely maintained in the communities, but animal husbandry has been significantly impacted by the emergence of packs of feral dogs, which attack birds, sheep, and calves. As a result, many families have chosen to stop raising animals (citation focus group 1, in Table 1).

Food heritage, understood as the knowledge and practices related to the production, collection, preparation, and consumption of food within Mapuche culture, is considered a facilitating factor, as this knowledge is still preserved. Older women are the ones who re-signify food heritage, noting that young women have discontinued this role, both in feeding their families and in educating their children. Likewise, institutions do not take responsibility for preserving this type of heritage knowledge either (quote from community leader 3, in Table 1). Family support is essential for older adults, but this does not mean they are unable to engage in activities related to their food sustenance. Having family support helps them maintain their food production units (quote from interview 2, in Table 1).

Community spaces are also important places for Mapuche culture. They are cultural territories where people can meet, carry out their domestic chores, and keep their livestock. They are spaces where water was frequently found and was accessible to all because the sites were not fenced. In these places, people conversed, played, and generated bonds of trust, in addition to the aforementioned tasks (multiple interview quote 1, in Table 1).

Family food production is considered essential for food security, as it ensures the availability of food defined as culturally healthy and accessible to families. The vegetable garden emerges as both a practical and symbolic element of great significance, directly linked to food security. It also fosters the development of cultural food practices and socialization among neighbors. Adult and elderly women are typically responsible for the daily tasks of maintaining and harvesting the garden, which is characterized by its diversity, including polycultures, seasonality, and the use of traditional knowledge for fertilization and pest control. Greenhouses have also been incorporated into production techniques. These systems enable bioavailability because they are considered healthy, chemical-free foods, particularly in terms of fertilizers and pesticides for vegetables and hormones for animals.

### 4.2. Food Safety Barriers

Regarding barriers to food security, the following codes or categories were identified: pathological old age, which refers to the perception of the elderly as sick, dependent on care, and unable to perform productive tasks. This perception is both social and cultural, based on the biomedical approach, and extends beyond self-perception and the family environment. It originates in the institutional sphere, influencing policies that focus solely on providing assistance to the elderly. This perception disrupts the cultural role traditionally assigned to the elderly, which includes being carriers of cultural wisdom and bearing the responsibility of passing down diverse knowledge, such as that related to food security, which ensures cultural survival for the community and future generations (multiple interview 1, in Table 2).

Living alone is a barrier, given the perception just indicated. The migration of young people and labor dynamics influence the elderly to be alone (interview 2, in Table 2). Water scarcity is caused by the deteriorated environment due to the loss of biodiversity and soil erosion. One of the fundamental causes referred to is the deforestation of native species and substitution with exotic species. In the case of the territory, there is also the contamination of water courses by the percolates from the Boyeco landfill. Families receive water, which is insufficient for their domestic needs and consequently does not allow the maintenance of vegetable gardens, animals, and poultry, which would contribute to the food security of the rural elderly, their family group, and the community (citation focus group 2, in Table 2).

The restrictive economy code refers to people’s perception of how institutions value the needs of rural families and the lack of knowledge about these needs, particularly those related to the elderly. This can be seen in the low budget of the programs aimed at them, which do not achieve results that have an impact on the quality of life of families (citation interview 4, in Table 2). The labor dynamics category refers to the impacts on the lives of farming families who have been forced to work outside the fields, mostly in the city of Temuco. The impacts are cultural, social, and economic. The dynamics of the people who are active in the labor force imply being away from home and the farm all day, which does not leave them time to carry out productive and collaborative tasks and allow food production by the elderly. It is also cultural and social because this dynamic prevents the continuation of collective tasks and productive support in the community, which ultimately breaks family, intergenerational, neighborhood, and community relationships (quote interview 3, in Table 2).

Regarding inadequate public policies, it is linked to the references in which the participants state that these are not adapted to the needs of the rural Mapuche elderly due to a profound lack of knowledge of the culture and the territory, in addition to a lack of intersectoral coordination of the public system, which translates into a lack of public policies with a productive/economic approach aimed at this age group (quote from community leader 2, in Table 2).

It is important to highlight the situation that, according to the interviewees, refers to the conservation and reproduction of the food heritage, which is part of the role assigned to women. Specifically, this role is observed in the activities of raising poultry and small animals, as well as in the production of healthy food in a family garden. Men can collaborate in this situation when they are older if their health situation allows it.

The barriers are basically identified with a brake on the cultural action that allows food security through the production of healthy food, to which environmental problems, cultural changes that destabilize group identity, and inadequate public policies due to ignorance and lack of commitment to the worldview of the inhabitants of the territory contribute, in addition to poor intersectoral coordination.

### 4.3. Socio-Cultural and Environmental Elements Associated with Food Security

The facilitating elements are the ancestral knowledge safeguarded by rural Mapuche elders and respect for cultural traditions regarding the production of food that allows and sustains their food security.

The impossibility of having food security in a culturally diverse territory is limited by the failure to establish horizontal intercultural relations that would imply knowledge and respect on the part of the dominant social structure towards the ethnic minority with which it relates. In this case, the dominant social structure is represented by the institutions mandated by the state to fulfill this function; however, by not knowing and valuing in depth the worldview of the vulnerable group, it is not possible to strengthen or promote food security in these territorial spaces, especially due to the added social prejudice towards the elderly.

Socio-cultural and environmental elements associated with food security are present in the discourse of the study participants, with which the following associated conceptual categories are constructed.

Among the socio-cultural and environmental elements associated with food security, the following codes or categories stand out, constructed on the basis of the discourse of the participants: In this conceptual network, an important element emerges which, being an environmental element, takes on great symbolic relevance in the socio-cultural sphere for the food security of Mapuche elders; this is community water of collective service and management. The code of loss of community life refers to the denial of cultural expression to which the Mapuche people have been subjected, translated into the cessation or reduction in family, productive, and community practices as a result of the assimilation to dominant culture (quote from focus group 2, in Table 3).

The food heritage category is understood as knowledge and practices related to the production, collection, and consumption of food based on the Mapuche cosmovision that support the concept of food security specific to this culture (quote from focus group 1, in Table 3).

Finally, the labor migration code for young people, which is linked to the references in which the participants indicate that the absence of young people who contribute to the work of agricultural production for the family and community generates food insecurity in the elderly due to the scarcity and replacement of food that is culturally recognized as healthy. This absence is caused by the economic needs of the family group since there are no economic and labor solutions in the rural sector. The main impact of this phenomenon is a weakening of community ties since this age group is excluded from community participation and, therefore, their families, particularly the elderly, as they are not part of the support for the productive tasks of their families or neighbors and, consequently, they cannot be subject to such support, which weakens community ties and food security.

Among the environmental elements that influence food security from the perspective of the interviewees are drought, feral dogs, environmental aggressors, and the availability of water (quote from community leader 1, in Table 3). The code drought alludes to an environmental factor that negatively influences food security since it is not possible to grow crops or have a good and healthy diet (quote from multiple interview 2, in Table 3).

Feral dogs arrived in the territory, especially after the opening of the landfill that operated from 1992 to 2016. Families coming from the urban area of Temuco are accustomed to abandoning their dogs in the rural sector, and the Boyeco territory has become one of these preferential spaces, which has had unfortunate impacts on the family economy and the food security of the elderly (quote from community leader 1, in Table 3).

The previous element is connected to what we refer to as environmental aggressors, which, according to the research participants, are a constraint on food security. These include factors such as the presence of a landfill in the territory, which has had a profound impact on the environment and agrifood production. Participants also mentioned the use of agrochemicals, which has led to soil degradation, and the intensive use of water. All of these factors have significantly contributed to the loss of biodiversity, thereby reducing food security (citation focus group 1, in Table 3).

The use of agrochemicals is highlighted by the participants because, according to their perspective, they deteriorate the quality of the soil, causing the loss of biodiversity, soil erosion, and a lack of water (citation focus group 1, in Table 3).

The code “Intensive use of water” is associated with the use of this element by productive projects external to the communities, which produces water scarcity within the communities and does not allow for vital and productive domestic reproduction. This is related to what is defined as the problem of “water availability”, which is linked to the references in which it is stated that not having good access to water is an important environmental factor that generates threats to food security (quotes from interview 2 and interview 1, in Table 3).

In this analysis, it is important to highlight that there is more than one sociological subject among the participants; that is, the group does not present characteristics of absolute homogeneity. Among them stand out those who promote and maintain their Mapuche cultural practices and the subjects that are constituted by people linked to the evangelical religion, who discontinue their cultural practices, but nevertheless, among their principles, maintain the cultural definition of the Mapuche cosmovision with the environment, which contributes to have related definitions of food security. However, this subject reinforces their cultural ties based on their religious ties, self-defined as brothers in the faith, differentiating themselves from other members of non-evangelical communities.

### 4.4. Conceptualization and Components of Food Security

The following codes or categories were identified in relation to the participants’ perception of the conceptualization and components of food security and its implementation in rural areas.

A central element in this conceptual framework is what is referred to as the conservation of food heritage, which in symbolic and cultural terms forms the foundation of human development, as food not only nourishes but also heals. This is reinforced by what participants refer to as good food, which comes from their own traditional crops, including animal husbandry. Therefore, the concept of food security is understood as the consumption of nutritious and healthy food produced by the family, which is also meaningful within their own cultural context (quote from community leader 2, in Table 4).

The concept of food security within the culture investigated contains the components of land quality and water availability, in addition to the abundance of traditional foods known as healthy foods. Packaged foods are negatively valued because they are processed and contain unhealthy substances. These are opposed to healthy food from family and local production and a way of life that interviewees call küme mogen, a good way of living, which not only defines individual behavior but also contemplates respectful interaction and conservation of the environment and non-human species. In this way, we protect, not damage, that which allows us to survive as a species, all of which, according to the participants, contributes to and safeguards food security (quotes from interview 3 and interview 1, in Table 4).

Finally, community solidarity is highlighted, which reinforces knowledge and heritage production practices, which constitute the basis of food security for a culture (quote from focus group 1, in Table 4).

The knowledge of the heritage of food security is safeguarded by the older people of the territory; however, the current socio-cultural situation, in which young people and adults work outside the territory, prevents this knowledge from being shared and reproduced within the culture (quote from focus group 1, in Table 4). In this area, it is worth highlighting the importance of the role of women in the conservation of the food heritage and, consequently, in the strengthening of the food security of families, communities and the Mapuche people; the vegetable garden, under women’s control, is an icon of family food security (quote from community leader 3, in Table 4).

In relation to the implementation of the concept of food security relevant to the territory and culture, from the perspective of the interviewed subjects, we mainly observed the need for family support for the performance in the daily life of the elderly so that they maintain and apply their knowledge in the practice of the production of food considered culturally healthy. This would be possible in the current socio-cultural and economic context through the support of state institutions for the generation of productive infrastructure necessary for food production, such as the recovery of the quality and health of the land and water management technologies, among others. A relevant and fundamental element, according to the cultural support referred to, is the existence of a vegetable garden for self-consumption, which is the element that provides permanent food security to rural Mapuche elders (interview 2, in Table 4).

To this end, it is considered essential to support participatory public policies that are based on listening to the people and their practical and cultural needs in order to solve local problems. According to those interviewed in this territory, it is important to develop the following initiatives aimed at the reforestation of natives to recover the fertility of the soil and maintain the water supply: multiculture projects, labor contracts to support infrastructure for the cultivation of vegetable gardens of the elderly, the improvement and rest of the land, community water harvesting to solve water shortages, and the use of organic fertilizers that allow the cultivation of healthy food and recover the health of the land (in Table 4).

Finally, the participants state that it is necessary to carry out actions aimed at recovering food heritage and reproducing knowledge both in Mapuche elders and in new generations. In this regard, they mentioned that currently, public institutions do not contribute to promoting its reproduction. Fundamental concepts for the definition of food security are food cultural heritage and what they call küme mogen, which are associated with good living among human beings, other species, and the environment.

## 5. Discussion

Different factors that influence the food security of rural Mapuche elders are observed. One of the elements that stands out is the role of cultural heritage in food production and consumption, since older people continue to promote the practice of traditional knowledge about crops and animal husbandry, which favors their food self-sufficiency. However, the migration of young people to the cities and the lack of structural support have weakened the communities’ capacity to maintain these practices.

One of the main barriers faced by these people is the limited availability of natural resources, especially water. Drought and pollution caused by human activities, such as the existence of the Boyeco landfill, negatively affect agricultural production and animal husbandry, restricting access to healthy and culturally appropriate food. This problem is aggravated by the lack of public policies consistent with the specific needs of these communities, which reflects a profound ignorance of the Mapuche cosmovision. The above evidences the scarce support of the public sector to the peasant and Indigenous sector, consistent with the analyses of the Observatory of the Right to Food of the University of Oviedo, 2019 [10].

On the other hand, a strong sense of cultural and community identity is identified as a key facilitator for food security. Older people value the use of traditional agricultural techniques and the transmission of intergenerational knowledge, although they recognize that these practices are at risk due to social and economic changes.

The impossibility of achieving food security in a territory with cultural diversity is limited by the inability to establish horizontal intercultural relations, which involve knowledge and respect from the dominant social structure toward the Indigenous people with whom it interacts. In this case, this structure is represented by institutions mandated by the State to fulfill this function through relevant public policies. However, since there is no deep understanding and appreciation of the worldview of the minoritized group, it is not possible to strengthen and promote food security in these territorial spaces, especially due to the added social prejudice and ageism against older adults.

This is how conventional agricultural production methods, which use agrochemicals in the implementation of prevailing public policies, negatively affect the perception of food security in elderly rural Mapuche people, as they contradict their concept of health in the human–nature relationship. Additionally, these practices violate the role of the rural elderly woman in traditional agriculture, who provides sustenance for the family, as is consistent with [11].

As a consequence, the State, in its interventions in Indigenous communities, has introduced a productivist paradigm based on chemical products and overexploitation of natural resources. In this context, the concern not only has to do with preserving the types of food and ways of cultivating, which for the elderly has an ancestral cultural value but also with the vulnerability in food security that these actions cause, with the lack of cultural relevance, since they are forced to consume food that they perceive as unhealthy. Likewise, in order to strengthen the food security of the rural Mapuche people, it is necessary to strengthen public policies in the use of technologies that allow for achieving the proposed objectives [31].

At the political level, it is essential to develop public policies that consider the Mapuche worldview and promote sustainable agricultural production in the rural context, as well as to overcome the ageism that these public policies entail when dealing with older people by implementing not only care-oriented actions for this age group but also productive and developmental actions, with age and cultural appropriateness. Initiatives aimed at improving access to water, rehabilitating land, and strengthening family production are also essential steps to ensure sustainable food security. Likewise, it is important to promote the active participation of communities in the creation of these policies to ensure that they are adapted to their realities and needs.

### Limitations of the Study

This study presents limitations that should be considered when discussing its results. An important aspect is that being a qualitative study with an ethnographic approach, despite being conducted over a large territory and representing nine Mapuche communities, the construction of generalizing theory requires comparison with results from studies in the area with other Indigenous peoples. According to [8], regarding the topic in Latin America and the Caribbean (LAC), there are only six related studies, with the greatest productivity found in North America, with 29 studies (United States and Canada), which could present comparative difficulties for theory construction, based on the inductive qualitative method used in this study. This limitation can be overcome by carrying out other comparative ethnographic studies, both nationally and in Latin America and the Caribbean, which would allow the construction of valid generalizations through the analytical use of the comparative inductive method.

The study was conducted during the period of 2022–2023, which may have influenced the results due to the specific circumstances of the previous years due to the pandemic. This may have exacerbated some problems related to food security, such as youth migration and access to basic resources, which does not necessarily reflect a long-term situation. Another limitation is the lack of quantitative data to support the results obtained; however, the qualitative approach allowed for an exploration of the perceptions and experiences of the participants. It could be said that the absence of numerical data limits the ability to measure the scope of the problems identified and to make numerical comparisons with other populations or periods, so a mixed study could perhaps have a greater scope.

It would be interesting and innovative to approach the same topic from an intergenerational perspective because it would contribute to the construction of knowledge with a greater number of analytical dimensions.

## 6. Conclusions

This study provides an analysis of the factors that influence food security among Mapuche, older people in rural areas of Temuco, Chile. The results reveal the importance of cultural heritage and ancestral knowledge in food production and consumption. Older people, particularly women, play a crucial role in preserving traditional agricultural practices, which are fundamental to their food self-sufficiency and well-being.

The components of the definition of food security are closely related to the food heritage of the culture in question. The food heritage of a culture is a central element in the symbolic interpretation of the concept of food security and is the foundation for distinguishing those foods that are considered healthy, nutritious, and contributing to general health. According to older people, these foods should not be missing from a diet in order to have a long and good quality of life.

Food security in these communities faces several barriers. Key among them are environmental degradation, a lack of adequate access to water, and the decline in the youth workforce due to migration to cities. These factors have reduced the ability of older people to maintain sustainable agricultural practices and ensure a nutritious and culturally appropriate diet. Furthermore, current public policies do not effectively respond to the specific needs of rural Mapuche communities, which increases their vulnerability.

The sociological subject to which these public policies for the Mapuche people or other Indigenous peoples could be directed is not homogeneous, nor unique, that is, the group in question does not present characteristics of absolute homogeneity, so universal actions, without territorial distinction, are not very pertinent, their differentiation is based on the cultural heritage of each people, and a common aspect is that their worldview has as its axis the link of respect and horizontality with the environment. Despite these challenges, cultural heritage remains a key enabler of food security. Traditional farming and animal husbandry practices, as well as the use of ancestral knowledge about biodiversity, continue to be valuable resources. However, to ensure the survival of these practices and improve food security in these communities, it is imperative that public policies are tailored to their cultural and territorial realities.

The findings suggest that while rural Mapuche elders retain valuable practices for their food security, inadequate policies, migration, and environmental degradation present significant challenges. Comprehensive political and social intervention is required to strengthen local resources and ensure the continuity of food traditions that are crucial to the health and well-being of this population.

## Figures and Tables

**Table 1:** Identification of food safety enablers.

Participants	Testimonials
Interview 4	“If they are cultivating in their fields they have the possibility of growing their own food, which is like the culture they grew up with, the food like the cereals that they sowed in the houses, the legumes, the vegetables, in that I think they do tell me security”.
Community leader 1	“The food is mainly soup, that’s like good food, lots of toasted flour, seasonal vegetables”.
Focus group 1	“It was also necessary to lock them up early, I left them and it was dark, and that was it! and of course the dogs got into the woods there and caught the sheep cornered, and they killed three sheep, they left in a shot”.
Community leader 3	“but there is also no accompaniment, so to speak, as to what food they can prepare with what they have in their homes, with what they can produce, or what they know culturally”.
Interview 2	“Before, life in the countryside was more relaxed, it was somehow more bearable. One did not live in this tension of having to fulfill schedules, we had more time to be able to dedicate to the old people, but not now poh, now the person who works has to be leaving already at 6 in the morning in the bus some 7 in the morning at least 7: 30 in the morning he or she has to be out of the house, so that means that the grandmother or grandfather has to get up alone in the morning and has to struggle alone and if he or she has little mobility then he or she will no longer be able to go to the garden to look for a product to make something healthy, but will have to eat what they left or what was left from the previous day”.
multiple interviews 1	“Before, it was nice to wash in the marshes because you washed and sometimes your clothes would come off, the streams like that, you don’t know that the only thing left there was the broom, or it was a bit funny to go far away to fetch water in a bucket or to go there to wash. I knew that the ladies would get married later (everyone laughs), not nowadays, nowadays everyone has a house so they can’t even go out to the countryside (laughs)”.

**Table 2:** Food safety barriers.

Participants	Testimonials
Multiple interviews 1	“The health of the old person has nothing to do with now, that is why there are many old people who have already reached eighty-four years old, ninety-two years old, even one hundred and five years old, because that is what they were fed with another type of food, with the food from before, natural. Nowadays no, the same food brings illness, it depends on how you eat, it depends on what you are eating, it produces illnesses, sipo (reaffirms) because if one for example, is eating fatty things every day, I don’t know, every day you add something fatty to the bread, in the end what does it all produce, gall bladder, you have to have a gall bladder operation, and in the old days the old people that one knew, when did they know about having a gall bladder operation?”.
Interview 2	“It’s not easy for them to ask for help, it’s not easy to say you know that I need this or suddenly they feel that they are abusing someone who is going to help them, and they prefer not to tell him anymore, because they feel it has been too much”.
Focus group 2	“But these streams do not respect [waters contaminated by a landfill], the streams follow their course, when the time of winter comes they overflow to all sides, and the water flows out, so they cannot say that there is no contamination. That estuary is called Cusaco, that is, there are two estuaries, in this case the one on this side, which is not very big, but the other one is bigger, so they come together and that estuary was analyzed and came out contaminated”.
Interview 4	“the Mapuche in this case, which is us, it cost us, it costs us because the Mapuche are always used to having a lot of things, but nevertheless, it has been decreasing more and more, today the greatest food security is within the subsidies that the old people receive, that we receive (because I am part of it too), in which we have to buy things and we cannot reproduce anything, because due to the lack of water and animals, we are practically just [starting] some families again”.
Interview 3	“In 1983 they begin to subdivide the lands, in which the families are left with a minimum extension of land that in this case Pinochet launches the law 2.568 where you have to subdivide the land and all the rest and many families of course agreed to this, even though there was some opposition to this law, but most of the communities in this place were subdivided and then the people began to fence their space and of course there was less space for animals and from there they began to subdivide, the people because they no longer, because in the past everything was collective there were spaces where they could install the animals and most of us had cows and oxen”.
Community leader 2	“Public policies from INDAP [Institute for Agricultural Development], from agriculture, insist on programs such as PDTI [Indigenous Territorial Development Program], PRODESAL [Local Action Development Program] and PRODER [Rural Development Program], where the only thing they explain to the people is that they have to produce and hopefully a lot (emphasis), and that a lot versus quality, in my opinion, does not make sense, but for the people, according to the economic aspect, what the economic income implies, when selling those products, it makes them reach these institutions and try to maintain the link with the institutions to produce better, in parenthesis, to produce better, that is what they call it (emphasis, raises his voice). Because that is how the trained technicians come, what do I know, the engineers, right, to tell him how the farmer has to produce today, mainly because in the past production was from here upwards, and today they come from there, from the academy, perhaps to teach how to produce already, and that is where this issue is mixed, they bring you contaminants versus what used to be produced in the past, which was healthy”.

**Table 3:** Sociocultural and environmental elements associated with food security.

Participants	Testimonials
Focus group 2	“A neighbor needs help and that’s it, he goes out to ask for help, but at the same time it’s hard for them to return the hand, which used to be normal. The one who was going to sow was made a mingaco all together, and then he had to give his hand back. And now, what happens now, is that we suddenly work with a tractor, we run out of time and what happened, that in the long run this has also made us to be more individual”.
Focus group 1	“I would separate them into two major food safety issues. One is the issue of healthy food, which is what we have talked about here, cultivating without agrochemicals, that if the tomato comes out deformed, it doesn’t matter because it will have a real flavor. Of course, if it has bugs, or apply as the ancients did the ash for pests, the apple cider vinegar water, how to apply all these techniques avoiding the same thing and preserving the seed. That is liberation, sovereignty. And on the other hand, to have economic autonomy, not, as Renato said, no, to have the need to have enough money to be able to obtain food, so that gives you economic autonomy, not having to go shopping, as little as possible”. Focus Group Jeronimo Melillan Community”.
Community leader 1	“The drought is something that has had a great influence, people still continue to make their farms, vegetable gardens, for self-consumption, as something that everyone tries to maintain as much as possible, because there are people who are already old and have some kind of illness and do not have good access to water”.
Multiple interviews 2	“Long time ago, when there were not so many eucalyptus trees, we had a water dam, we used to irrigate with a water motor where I was telling you, and we used to irrigate with a water motor, and now, even if you have a motor, you have nowhere to get water, that is the problem”.
Community leader 1	“There was a big issue here a while ago with the landfill and the arrival of many dogs, but even so, they still continue to arrive, and suddenly they will kill their chickens and other poultry that they have in the house. They still continue to arrive and suddenly they are going to kill their chickens and other poultry that they have in the house, as well as sheep. Little by little, people have been raising poultry and smaller animals again”.
Focus group 1	“Yes, we had a landfill for many years here, very close by, it contaminated the water table and the animals got sick, the children, who were used to bathing and drinking water from the river, also got sick, yes, we were there. The trees are no longer the same…before autumn arrives they turn yellow, but as well as with scale, many native trees began to burn, because of the focus of the landfill, for some reason it was also closed because it was there for many years, how long was it there for 18 years? 25 years! So there was a lot of pollution and that affected everything because we are right with the north wind and it took everything to this sector”.
Interview 2	“The foods that were eaten before were of frequent use and valuable for us, the bloodroot they called it, also the watercress of the swamps were taken and consumed and were exquisite and that was eliminated and ended product of the chemicals”.
Interview 1	“There are people who are already old and have some kind of disease and do not have good access to water, so for them it is very complex and they try to take advantage of this rainy weather to have something and try to maintain this type of food”.

**Table 4:** Conceptualization and components of food security and its implementation in rural areas.

Participants	Testimonials
Community leader 2	“[FEEDING] is part of the way of life, it is part of the lawen, it is part of good living, it is part of human relations, of the inter-species relation, that is to say, feeding is not only throwing food in the mouth. It is the relationship of this body, which is not only material, but also spiritual, the relationship, the interaction with nature, the interaction with the world, with the other species, basically, it is the good living associated to harmony, not to overeat, not to undereat, not to overeat, not to undereat”.
Interview 3	“Security means that nothing is lacking and that hopefully in rural areas we can have a permanent surplus [of] food, which is what we see today is not happening because generally families look for resources outside the community to be able to feed themselves and bring in food from outside. And especially in young families because there is no longer a productive issue, especially in the Boyeco territory, due to water scarcity, water contamination and also because people have left aside (the Mapuche in this case) the issue of natural production because the animals that produce guano are no longer present”.
Interview 1	“Food safety we could even say to protect what we grow and what we have by not adding chemicals to it, so that it can make a moderately safe food for us”.
Focus group 1	“A neighbor told me that in the past, I don’t know, people used to get together to fix the roads, it was because people lived here, they had the time and were the owners of their time, for example when one sowed wheat, they had to, they had to prepare the land at a certain time of the year, in a certain week of the month, then sow, then nothing is done until the harvest, and so, in between, they were doing other activities, for example picking peas, others were dedicated to breeding, but the people lived and worked here”.
Focus group 1	“I tell him a lot, for example, a child was learning as he was watching the adult, and as the adult is now working in the city, the children do not learn”.
Community leader 3	“That is what makes them feel good and they feel proud because I don’t know, for example, the old ladies visit each other and what they see first is the vegetable garden, the gardens are shown and they invite each other, for example, the first new parents come out and visit each other and take them to their relatives or neighbors and they invite each other, the other brings back something else, for example, new beans or peas. And so on and so forth, that is the way of life that is slowly coming back, because there was a time that had definitely been lost”.
Interview 2	“The PACAM [Programa de Alimentación Complementaria del Adulto Mayor] that is given to the elderly is a tremendous contribution, it is a contribution for those who have nothing, but it cannot be the solution, it is a patch. But what they should do is try to promote appropriate vegetable gardens for the elderly, that the family gets together, I don’t know, other things, networks for the communities, facilitate everything that is necessary”.
Interview 2	“The government should be more involved in the implementation of these projects to the communities and it should not be so bureaucratic to have to do a lot of paperwork to be able to apply for a small pool of water”.

## Data Availability

The original contributions presented in the study are included in the article, further inquiries can be directed to the corresponding author.
